# HT-Net: A Hybrid Transformer Network for Fundus Vessel Segmentation

**DOI:** 10.3390/s22186782

**Published:** 2022-09-08

**Authors:** Xiaolong Hu, Liejun Wang, Yongming Li

**Affiliations:** College of Information Science and Engineering, Xinjiang University, Urumqi 830000, China

**Keywords:** fundus vessel segmentation, Transformer, CNN, HT-Net

## Abstract

Doctors usually diagnose a disease by evaluating the pattern of abnormal blood vessels in the fundus. At present, the segmentation of fundus blood vessels based on deep learning has achieved great success, but it still faces the problems of low accuracy and capillary rupture. A good vessel segmentation method can guide the early diagnosis of eye diseases, so we propose a novel hybrid Transformer network (HT-Net) for fundus imaging analysis. HT-Net can improve the vessel segmentation quality by capturing detailed local information and implementing long-range information interactions, and it mainly consists of the following blocks. The feature fusion block (FFB) is embedded in the shallow levels, and FFB enriches the feature space. In addition, the feature refinement block (FRB) is added to the shallow position of the network, which solves the problem of vessel scale change by fusing multi-scale feature information to improve the accuracy of segmentation. Finally, HT-Net’s bottom-level position can capture remote dependencies by combining the Transformer and CNN. We prove the performance of HT-Net on the DRIVE, CHASE_DB1, and STARE datasets. The experiment shows that FFB and FRB can effectively improve the quality of microvessel segmentation by extracting multi-scale information. Embedding efficient self-attention mechanisms in the network can effectively improve the vessel segmentation accuracy. The HT-Net exceeds most existing methods, indicating that it can perform the task of vessel segmentation competently.

## 1. Introduction

The eyes can obtain approximately 80% of external information, which is unmatched by other human organs, so healthy vision is essential for humans. Nowadays, as people work more intensively while ignoring eye protection, more and more young people are suffering from eye diseases. People are increasingly relying on medical technology with the advent of digitalization. In the past, the ways in which doctors treated patients, through their own experience, gradually shifted to a reliance on high-precision medical instruments, so accurate disease data became crucial.

For ophthalmic diseases, doctors can obtain a wealth of pathological information from the imaging of fundus blood vessels. For example, partial swelling of fundus blood vessels can usually be diagnosed as diabetic retinal disease [1]. Increased curvature and narrowing of vessels can be interpreted as a hypertensive retinal disease [2]. In addition, high-quality vessel images can also be effectively applied to cellular ophthalmology, which is a crucial stage of cellular ophthalmic research.

The microvessels in the fundus vessels are uneven in width and extremely fragile, making them prone to missing and breaking during segmentation. The contrast of the overall image is low, and definition of the edge of the blood vessel is not apparent, which increases the difficulty of high-precision blood vessel segmentation. High-quality fundus images are essential for subsequent vessel extraction. Fundus images are generally obtained through instruments such as ophthalmoscopes, optical coherence tomography, and fundus color cameras [3]. In addition, experienced experts obtain accurate vessel maps by manually labeling the vessel pixels in images, which is challenging. Therefore, it is of great significance for doctors to find a fast and accurate method for the automatic segmentation of targets.

In recent years, the convolutional neural network (CNN) has significantly changed deep learning, with its excellent feature representation capabilities, which have extensively accelerated the progress of professionals in fields such as image recognition [4], image segmentation [5], and object detection [6]. The convolution operation is a method for local information extraction based on neighbourhood pixels. Deep learning models aggregate globally by overlaying multiple convolutional and pooling layers. Currently, the model based on the encoder–decoder architecture has contributed considerably to medical imaging, among which U-Net [7] is the most classical. Since most medical datasets are small samples and the structural features are relatively simple, U-Net performs better in medical image segmentation with a shallow network depth. Nowadays, more and more U-Net-based variant methods are being proposed and used in medical image segmentation, such as DUNet [8] and MAU-Net [9]. However, some CNN-based U-Net variants [8,9] are still deficient. Firstly, the convolutional operation only collects information from neighboring regions and lacks long-range dependency. Secondly, the U-Net variants still suffer from low accuracy in segmenting microvessels in fundus images. Finally, some of the microvessels in the vessel maps obtained by some U-Net variants can have breaks. Based on the above issues, this paper designs a hybrid Transformer network (HT-Net) for the segmentation task, a variant of U-Net. The main contributions include the following: (1) as the local information at the shallow level of the network is critical to medical image processing, we propose a feature fusion block (FFB) to enhance its feature representation capability by increasing the complexity of the block; (2) as there are many blood vessels of different scales distributed in fundus images, we propose a feature refinement block (FRB) that overcomes the problem of the varying scale of blood vessels by extracting multi-scale information; (3) due to the limitations of the convolutional operation itself, a hybrid CNN and Transformer network is proposed to realize long-range information interaction.

We propose a hybrid CNN and Transformer architecture for the retinal vessel segmentation task. An efficient self-attention mechanism is used for the vessel segmentation in this network, and global dependencies are established. In addition, the novel FFB and FRB are proposed, which are outstanding for the detection of microvessels in fundus images. The following section describes the HT-Net and related modules in detail.

The second part of the paper introduces the relevant literature. The third part describes the HT-Net and its blocks. The fourth part focuses on the relevant metrics and datasets. The fifth part validates the effectiveness of the HT-Net through multiple sets of experiments. The sixth section concludes the paper.

## 2. Related Work

In this section, the paper introduces unsupervised and supervised methods. The fundus vessel extraction network based on deep learning is a typical binary classification model that classifies detected vessels as 1 pixel and the background as 0 pixels before subsequent processing.

### 2.1. Unsupervised Methods

Unsupervised segmentation methods generally require an expert to process the data according to their characteristics and design feature extractors manually or semi-manually.

Tao et al. [10] proposed a superpixel-based fast fuzzy c-means clustering algorithm, which helps to integrate adaptive neighboring information and reduce the number of different pixels in a color image. Then, a sample color histogram computational method is used to achieve color image segmentation. Tang et al. [11] proposed an algorithm called patch-based fuzzy local similarity C-means, which can well characterize the relationship between image pixels $V_{i}$ and clustering center $V_{k}$. Wang et al. [12] proposed a segmentation algorithm that does not require pre-processing or training. The method uses matched filtering to enhance the vessels and then performs a hierarchical decomposition of the enhanced fundus images to achieve precise localization of the vessels while removing noise. Yin et al. [13] proposed a vessel tracking method that uses local grey-scale information to optimize vessel edges and then uses a Bayesian approach to identify the spatial structure. Qin et al. [14] proposed a segmentation method based on multi-scale information analysis and adaptive thresholding, which can detect vessel information at different scales.

In addition, expert analysis of the dataset and the design of a specific feature extractor are key steps in unsupervised methods. As a result, the method has poor generalization performance.

### 2.2. Supervised Methods

In contrast, supervised methods require a dataset with ground truths for training. The supervised methods have succeeded in the image field, mainly because CNN uses convolution operators. The convolution operation gathers local information from the neighborhood pixels and filters the image using a sliding window. The local connection and weight sharing in convolution operations effectively reduce the computational complexity and number of parameters. However, the convolutional operation cannot effectively capture the long-range dependency. It only expands the receptive field by stacking multiple convolutional and down-sampling layers.

Deep learning-based fundus image segmentation has developed rapidly and achieved excellent results. Ronneberger et al. [7] proposed U-Net, which is effective in processing medical images. Due to the excellent performance of U-Net in the field of medical image segmentation, more and more scholars have proposed variants based on U-Net and used them for medical image processing. Zhuang et al. [15] proposed LadderNet by improving U-Net, which consists of multiple encoder–decoders with skip connections between adjacent encoders and decoders at each layer. Wang et al. [16] proposed DEU-Net, which uses two parallel branches in the network’s encoder to collect spatial and more semantic information. Yue et al. [17] proposed a U-shaped structured network for fundus vessel segmentation, achieving spatial context enrichment by embedding multi-scale input layers and dense blocks in a conventional U-Net. Guo et al. [18] proposed SD-UNet. The dropout is replaced by a structured Dropblock in the basic convolution block of the SD-UNet, which effectively prevents overfitting. Guo et al. [19] proposed the lightweight SA-UNet. The main contribution of SA-UNet is the adaptive refinement of features after embedding spatial attention into SD-UNet.

Currently, scholars are introducing the visual Transformer [20] into the field of medical image processing. The self-attention mechanism in the Transformer is a computational principle that achieves long-range dependencies through a contextual aggregation mechanism. Xu et al. [21] proposed a lightweight Transformer model that captures local–global information, improving precision and reducing the number of operational parameters. Huang et al. [22] proposed a dual encoder network that includes a global and local encoder, enabling long-range information interaction while collecting detailed information. Karthik et al. [23] proposed a practical 3D non-local attention module, which can learn comprehensive attention functions so that a voxel’s receptive field is not restricted to its local spatial neighborhood but in the global context. We can apply such an idea to the task of fundus vessel segmentation, where pixel features are tuned to respond to the global fundus image for enhanced feature representation.

At present, more and more scholars are applying Transformers to medical imaging. However, there are limitations in the training of Transformer networks. Firstly, when the input to the network includes large-resolution images, the self-attention causes a considerable training and inference overhead when operating. Second, Transformers do not have inductive bias for images, making it challenging to train models with small samples of images [24]. Therefore, researchers set the input model’s images to a small resolution, such as 16 × 16 patches as the input sequence [24], but this is still not perfect. Gao et al. [25] proposed an improved self-attention mechanism, which can effectively reduce the computational complexity.

## 3. Methods

This section details the HT-Net and its components. HT-Net overcomes the lack of long-range dependencies for convolutional operations by embedding an efficient self-attention mechanism (ESM) in the deepest layer of the model. In addition, two generic novel modules are included in the HT-Net.

The well-known U-Net inspires the HT-Net network. The HT-Net backbone consists of an encoder–decoder connected by skip connections to compensate for the information lost due to sampling. The difference between the HT-Net and U-Net is that the former performs three down-sampling and up-sampling steps, while the latter performs four down-sampling and up-sampling steps.

**Figure 1:**
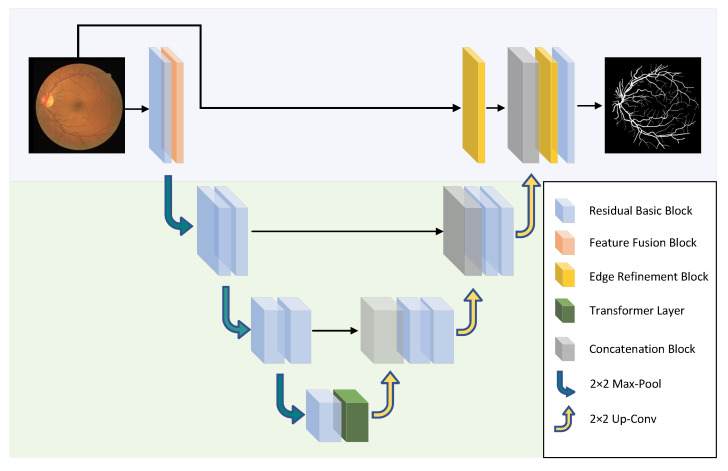
The schematic diagram of the HT-Net structure. The network uses 2 × 2 max-pool and bilinear interpolation for up-sampling and down-sampling.

**Figure 2:**
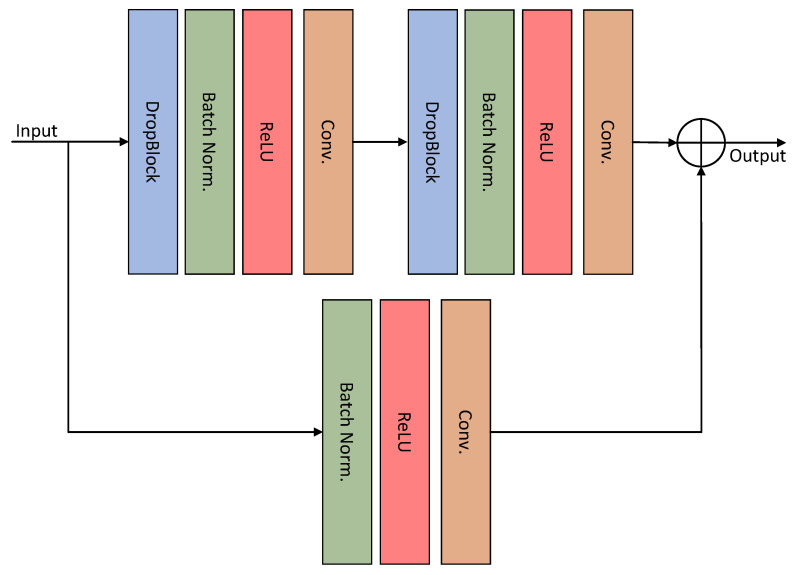
Structure of the residual basic block.

The schematic diagram of the HT-Net structure is shown in Figure 1. The first layer consists mainly of residual basic blocks (RBB) and FBB for the encoder section. The second and third layers are composed of a cascade of two identical RBBs. The fourth layer comprises a cascade of RBB and Transformer layers. The architecture of the RBB is shown in detail in Figure 2. Dropblock [26] is a regularization module modified from dropout to prevent overfitting by disconnecting the semantic associations between adjacent pixel regions. The FRB refines the image of the input network to obtain a feature map, which is then fused with the corresponding feature in the decoder. The following section will detail the FFB, FRB, and ESM.

### 3.1. Feature Fusion Block

The receptive field is one of the essential concepts in deep learning. If the receptive field is too small, the network can only observe the local features of the image. If the receptive field is too large, it usually contains much invalid information, although it has a more robust understanding of the global information. In order to improve the effective receptive field and avoid redundant information, capturing multi-scale features is a method commonly used by current researchers.

**Figure 3:**
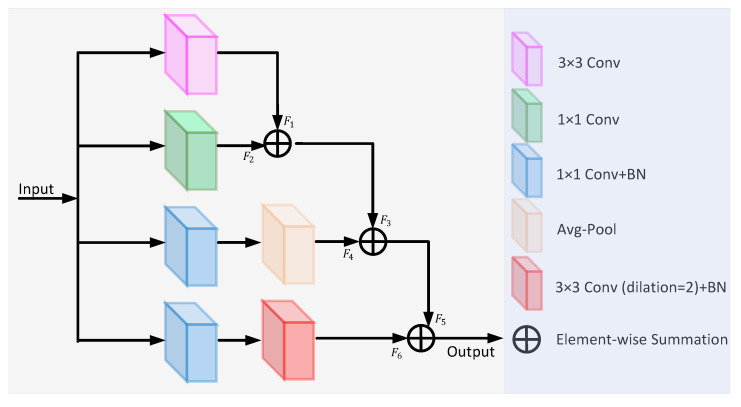
The architecture of the feature fusion block.

Inspired by the diverse branch block [27], we propose a feature fusion block (FFB), which enriches the feature space with four branches of multi-scale and complexity, including a convolution sequence, multi-scale convolution, and average pooling. The architecture of the FFB is shown in Figure 3. The different feature information is extracted by different branching sequences and then fused. The first branch consists of a 3 × 3 convolution operation and the output feature map $F_{1}$. The second branch uses a 1 × 1 convolution operation to add nonlinear features. The third branch consists of a 1 × 1 convolution, batch normalization, and average pooling, with the output feature map $F_{4}$. The fourth branch consists of a 1 × 1 convolution, batch normalization, and 3 × 3 dilated convolution with a dilation rate of 2. The feature output from each branch is fused one by one to obtain the final feature map.

In medical imaging, texture and edge information is generally extracted from shallow network positions, while global information is obtained from the deeper layers. FFB can accurately detect or segment objects by fusing feature information of different scales. Some fragile microvessels that are often missed can also be seen. Dilated convolution [28] can increase the receptive field to achieve multi-scale feature extraction.

### 3.2. Feature Refinement Block

For retinal vessel segmentation, ophthalmologists have access to well-structured and accurate images of the fundus vessels to reduce misdiagnosis during clinical diagnosis.

**Figure 4:**
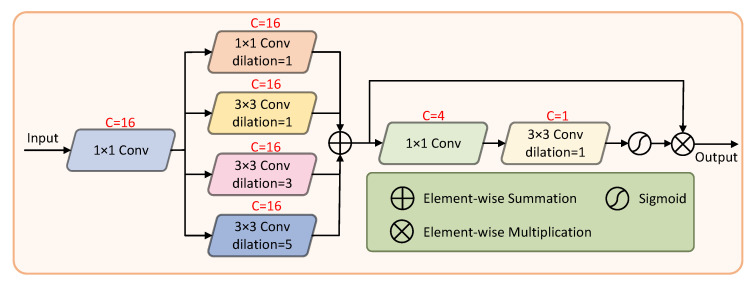
The architecture of the feature refinement block.

We propose a feature refinement block (FRB) to refine the edge structure of blood vessels and raise the accuracy. The structure of the FRB is shown in Figure 4. The input is a 3-channel pre-processed fundus image, followed by a 1 × 1 convolution operation to increase the dimensions of the images. The FEB uses a set of compact dilated convolutions to extract multi-scale information. Each branch collects feature information of different scales and finally learns rich edge features through feature fusion. Subsequently, a spatial attention mechanism [29] eliminates background noise to some extent. The number of channels is reduced by 1 × 1 convolution, and then 3 × 3 convolution is performed to generate a single-channel feature map. Finally, a sigmoid function is used to generate the attention descriptors. The fused feature map is multiplied with the attention descriptor to obtain the output.

### 3.3. Efficient Self-Attention Mechanism

Introducing self-attention into medical image segmentation can pose several challenges. For example, inputting a large-resolution image will increase the parameters, while inputting a segmented small-resolution image will destroy the integrity of the image to a certain extent. We address this issue by invoking an efficient self-attention mechanism (ESM).

**Figure 5:**
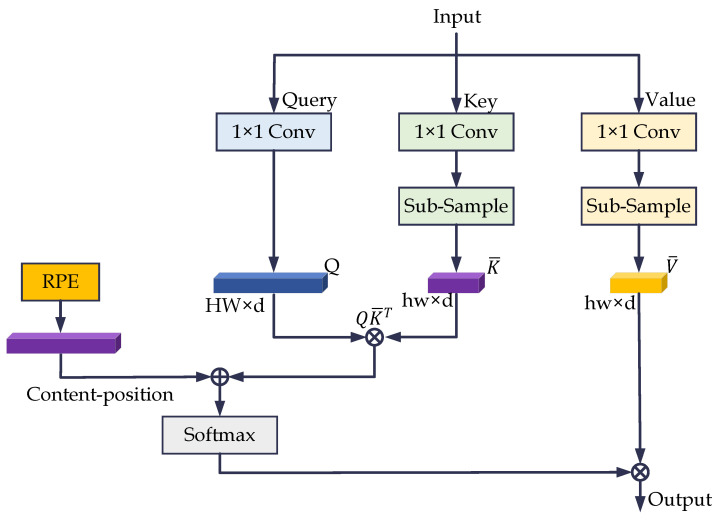
The architecture of the efficient self-attentive mechanism. RPE is the relative position code in the figure.

Since convolutional operations cannot capture long-range dependencies, we embed a Transformer layer with ESM in the bottom of the HT-Net. The architecture of the ESM is shown in Figure 5. The primary idea is to map the Query (Q), Key (K), and Value (V) with the projections, where $Q,K,V\in R^{n\times d}$, n=H×W. The dimensionality of K and V is then reduced by down-sampling to obtain $\bar{K}$ and $\bar{V}$, where $\bar{K},\bar{V}\in R^{k\times d}$, k=h×w≪n. *h* and *w* are the sizes of the subsampling feature maps. The formula for ESM is as follows.

(1) $$\mathrm{Attention}\left(Q,\bar{K},\bar{V}\right)=\mathrm{softmax}\left(\frac{Q{\bar{K}}^{T}}{\sqrt{d}}\right)\bar{V}$$

We used 1 × 1 convolution and bilinear interpolation for feature mapping and down-sampling. The down-sampled image resolutions for the DRIVE, CHASE_DB1, and STARE datasets were 37 × 37, 11 × 11, and 63 × 63, respectively.

### 3.4. Loss Function

We combine BCELoss and DiceLoss as our loss function. BCELoss performs well for binary classification tasks. Otherwise, the vessel pixel area only occupies a small part of the whole image, while the background pixels occupy the majority. We address the problem of data imbalance in fundus images by introducing DiceLoss. The overall loss function can be defined as follows.

(2) $${\mathrm{Loss}}_{total}={\mathrm{Loss}}_{bce}+{\mathrm{Loss}}_{\mathrm{dice}\;}$$

(3) $${\mathrm{Loss}}_{bce}=\frac{1}{N}\sum_{i=1}^{n}\left[y_{i}\log\left(p_{i}\right)+\left(1-y_{i}\right)\log\left(1-p_{i}\right)\right]$$

where *N* is the total number of samples, $y_{i}$ is the category of the *i*th samples, and $p_{i}$ is the predicted value of the *i*th samples.

(4) $${\mathrm{Loss}}_{\mathrm{dice}\;}=1-\frac{2|A\cap B|}{\left|A|+|B\right|}$$

where |A∩B| denotes the common elements between A and B sets, and |A| and |B| denote the number of elements of A and B, respectively.

## 4. Datasets and Metrics

### 4.1. Fundus Image Dataset

**Figure 6:**
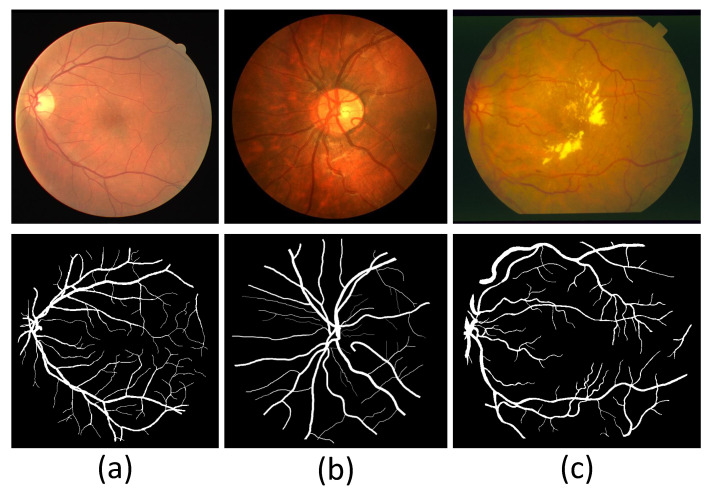
Sample images of three fundus image datasets. (**a**) A sample image of DRIVE. (**b**) A sample image of CHASE_DB1. (**c**) A sample image of STARE.

The experimental section of this paper utilizes DRIVE [30], CHASE_DB1 [31], and STARE [32] to evaluate the segmentation performance of the model. The three datasets contain the original images and corresponding labels, which experts manually label. DRIVE consists of 40 images with a resolution of 565 × 584, with a half split between training and test images. CHASE_DB1 includes fundus images with a resolution of 999 × 960. CHASE_DB1 has 20 training images, and the rest are test images. STARE contains 20 color images at a resolution of 700 × 605, 10 of which have pathological features. In addition, the STARE provider does not divide the training and test sets, so the user should actively divide them into the training and test sets. Figure 6 shows example images of the three datasets.

### 4.2. Dataset Pre-Processing

**Figure 7:**
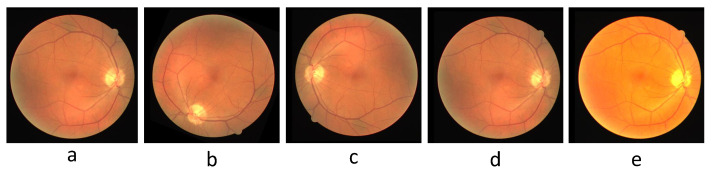
The pre-processed sample image of DRIVE. (**a**) Original image, (**b**) rotated image, (**c**) flipped image, (**d**) image after adding Gaussian noise, (**e**) image after color jittering.

There is a severe risk of overfitting when the dataset is fed directly into the model for training, so augmenting the dataset is a critical step before training the model. For DRIVE, we augmented the number of images by random rotation, color jitter, and adding Gaussian noise and flipping. The color jitter includes adjustments to contrast, brightness, and hue, and the flips include horizontal, vertical, and diagonal. Figure 7 shows an example image of pre-processing for DRIVE. For CHASE_DB1, we use the same data pre-processing approach as for DRIVE. The original image resolution of CHASE_DB1 is too large for proper training, so one original image is cut into four 512 × 512 images. Random rotations and flips augment the number of datasets for STARE. As the original resolutions of DRIVE and STARE are not suitable for the model, the image resolutions are adjusted to 592 × 592 and 704 × 704, respectively.

## 5. Results and Discussion

There were no additional pre-treatment or post-treatment steps for the experiments. The experiments were run on an NVIDIA TITAN RTX-24GB, with Adam as the optimizer and PyTorch as the framework. We set the training epochs to 70, 50, and 60 for DRIVE, CHASE_DB1, and STARE, respectively, set the batch size to 2, and set the learning rate to 0.005 uniformly.

### 5.1. Ablation Experiments

We propose a U-shaped HT-Net for retinal vessel segmentation. The network adopts a combination of CNN and Transformer for long-range information interaction. The model improves the accuracy and refines the microvessel structure by embedding a novel FFB and FRB. To analyze the performance of the ESM, FFB, and FRB for fundus blood vessel segmentation, we conduct ablation experiments in three datasets. Table 1, Table 2 and Table 3 show the results of ablation experiments on three public fundus datasets.

The results of the ablation experiments based on the three datasets show that embedding ESM in the backbone can considerably raise the overall segmentation performance due to the ability of ESM to capture long-range dependencies. We embedded both ESM and FFB in the backbone network to analyze the contribution of FFB to performance. The accuracy, F1 score, and AUC of the model after adding FFB are improved from the ablation experiments on the three datasets. The FFB is embedded in the model to extract information such as edges and textures, which are crucial for fundus images with relatively simple content. In the ablation experiments on DRIVE and CHASE_DB1, the performance was improved overall with FRB embedding. FRB gathers multi-scale information through dilated convolutions and then relies on an attention mechanism to focus on the more critical pixels and eliminate noise to a certain extent. Finally, the ablation experiments show that the results of our proposed HT-Net are optimal. The Acc, F1, and AUC in DRIVE, CHASE_DB1, and STARE reached 97.00%/82.79%/98.72%, 97.45%/81.54%/98.98%, and 97.65%/84.59%/99.28%, respectively.

### 5.2. Visual Analysis

**Figure 8:**
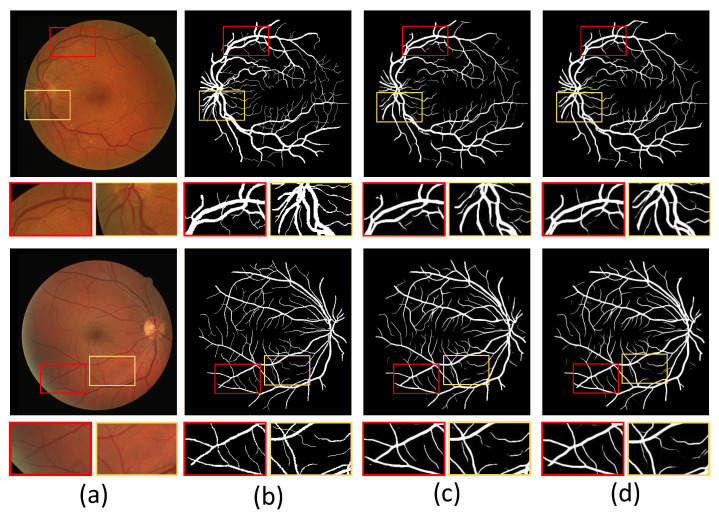
The example images based on DRIVE. (**a**) Original image; (**b**) ground truth; (**c**) visualization of U-Net; (**d**) visualization of HT-Net. The small patches are magnifications of the corresponding areas in images.

**Figure 9:**
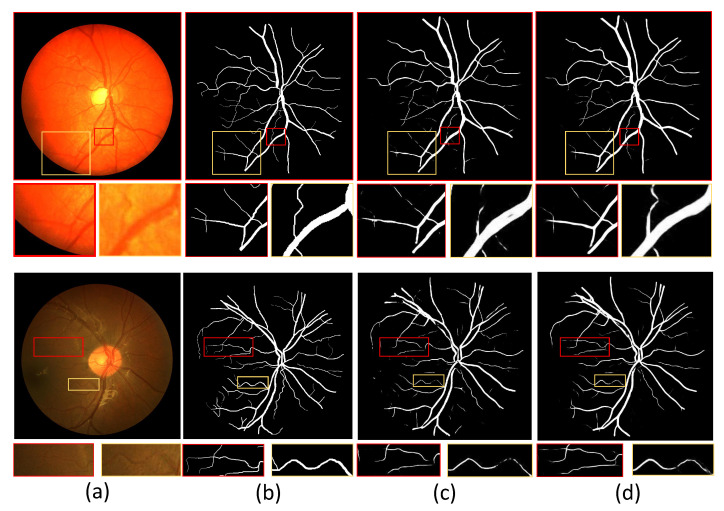
The example images based on CHASE_DB1. (**a**) Original image; (**b**) ground truth; (**c**) visualization of U-Net; (**d**) visualization of HT-Net. The small patches are magnifications of the corresponding areas in images.

**Figure 10:**
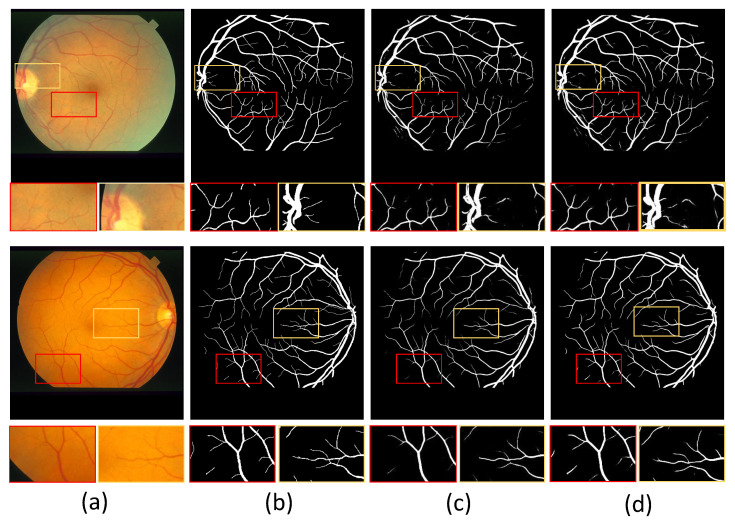
The example images based on STARE. (**a**) Original image; (**b**) ground truth; (**c**) visualization of U-Net; (**d**) visualization of HT-Net. The small patches are magnifications of the corresponding areas in images.

In addition, we show example images of test results based on the three fundus image datasets. Figure 8, Figure 9 and Figure 10 show example images of DRIVE, CHASE_DB1, and STARE test results, respectively, including the original, ground truth, backbone, and HT-Net networks, respectively. As shown in Figure 8, the example image of the backbone shows a broken vessel at the intersection of the different scales and the disappearance of microvessels. Compared to the backbone, the HT-Net example image improves segmentation at the intersection and substantially alleviates vessel breakage. For Figure 9 and Figure 10, the quality of HT-Net is significantly improved compared to the example images of the backbone. The HT-Net example image shows clearer microvessels and fewer broken microvessels.

With the naked eye, the quality of the visualized image of CHASE_DB1 is not significantly improved compared to the other two datasets. The main reason may be that the images’ low contrast makes the segmentation more difficult. Although visual analysis is intensely subjective, it also demonstrates HT-Net’s effectiveness for fundus vessel segmentation to some extent.

### 5.3. Comparative with State-of-the-Art Methods

To prove the effectiveness of HT-Net for fundus vessel segmentation, we compared the performance of several other high-quality algorithms on the three published datasets. Table 4, Table 5 and Table 6 are the performance comparisons with other advanced algorithms on DRIVE, CHASE_DB1, and STARE. The results show that the HT-Net achieved the highest F1 on all datasets.

The highest score for F1 of the HT-Net shows that the network can segment the background and blood vessels in fundus images better than other networks. The sensitivity of HT-Net was highest on CHASE_DB1 and STARE, which indicated that the model has a superior power ability in detecting vessel pixels. HT-Net did not achieve the highest sensitivity on DRIVE but outperformed most other methods. The highest accuracy and AUC were achieved for HT-Net on DRIVE (Acc:97%, AUC:98.72%) and STARE (Acc:97.65%, AUC:99.28%). In addition, the specificity of HT-Net is not optimal but comparable, demonstrating that HT-Net also performs well for the background. In conclusion, the HT-Net surpasses the most advanced models in retinal vessel segmentation.

### 5.4. Cross-Evaluation

A good retinal vessel segmentation model performs well on a specific test set and other datasets without fine-tuning. Table 7 shows the cross-training experiments of the HT-Net on the DRIVE and STARE datasets. The SP, ACC, and F1 are all first in absolute value when training on STARE and testing on DRIVE. Since the number of vessels is small and the images contain some lesions in the STARE dataset, training on the STARE dataset will make the model less capable of detecting thin vessels and more capable of detecting the background, thus decreasing the SE and increasing the SP. In the reverse case, a similar situation was observed. Training on DRIVE and testing on STARE results in higher SE and lower SP because using DRIVE as a training set enhances the ability of the model to detect blood vessels and decreases the ability to detect the background. Compared with other models, HT-Net performed the best in the cross-evaluation, showing that the model has a better generalization ability for retinal vessel segmentation.

## 6. Conclusions

We propose an HT-Net model, a fundus vessel segmentation network. HT-Net adopts a CNN at shallow levels of the network to collect local information. We combine the CNN with a self-attention mechanism in the deep levels of the network to capture long-range dependencies for more semantic representations. In addition, we propose two core modules, the FFB and FRB. The FFB boosts representation by increasing the complexities of the block. The FRB fuses the features from different dilated convolutions to obtain multi-scale information and then focuses on the critical pixels through an attention mechanism. We evaluated HT-Net on publicly available fundus image datasets (DRIVE, CHASE_DB1, STARE) and the results showed that HT-Net was superior to other networks in vessel segmentation. In future work, we believe that HT-Net may be used for other medical tasks, such as vessel and multi-lesion classification.

## Figures and Tables

**Table 1:** Ablation experiments based on the DRIVE dataset.

Methods	Se	Sp	Acc	F1	AUC
U-Net	0.7943	0.9833	0.9666	0.8059	0.9835
“ESM” only	0.8257	0.9824	0.9686	0.8209	0.9859
“ESM”+“FFB”	0.8204	**0.9840**	0.9696	0.8247	0.9869
“ESM”+“FRB”	**0.8431**	0.9810	0.9688	0.8252	0.9868
HT-Net	0.8256	0.9839	**0.9700**	**0.8279**	**0.9872**

**Table 2:** Ablation experiments based on the CHASE_DB1 dataset.

Methods	Se	Sp	Acc	F1	AUC
U-Net	0.8134	**0.9843**	0.9730	0.8005	0.9870
“ESM” only	**0.8572**	0.9813	0.9731	0.8092	0.9889
““ESM”+“FFB”	0.8493	0.9825	0.9737	0.8111	0.9894
“ESM”+“FRB”	0.8512	0.9821	0.9735	0.8103	0.9893
HT-Net	0.8477	0.9834	**0.9745**	**0.8154**	**0.9898**

**Table 3:** Ablation experiments based on the STARE dataset.

Methods	Se	Sp	Acc	F1	AUC
U-Net	0.8151	**0.9887**	0.9756	0.8350	0.9908
“ESM” only	0.8311	0.9876	0.9757	0.8390	0.9922
“ESM”+“FFB”	0.8471	0.9871	0.9764	0.8447	0.9928
“ESM”+“FRB”	**0.8571**	0.9748	0.9747	0.8379	0.9923
HT-Net	0.8478	0.9870	**0.9765**	**0.8459**	**0.9928**

**Table 4:** Comparison experiments based on DRIVE.

Methods	Se	Sp	Acc	F1	AUC
U-Net [7]	0.7943	0.9833	0.9666	0.8059	0.9835
DUNet [8]	0.8039	0.9804	0.9576	-	0.9821
LadderNet [15]	0.7856	0.9810	0.9561	0.8202	0.9793
DEU-Net [16]	0.7940	0.9816	0.9567	0.8270	0.9772
SA-UNet [19]	0.8212	0.9840	0.9698	0.8263	0.9864
PCANet [33]	0.8204	**0.9844**	0.9700	0.8274	0.9866
IterNet [34]	0.7735	0.9838	0.9573	0.8205	0.9816
MAU-Net [9]	0.7890	0.9799	0.9557	0.8192	0.9774
SCS-Net [35]	**0.8289**	0.9838	0.9697	-	0.9837
HT-Net	0.8256	0.9839	**0.9700**	**0.8279**	**0.9872**

**Table 5:** Comparison experiments based on CHASE_DB1.

Methods	Se	Sp	Acc	F1	AUC
U-Net [7]	0.8134	0.9843	0.9730	0.8005	0.9870
DUNet [8]	0.7779	0.9864	0.9653	-	0.9855
LadderNet [15]	0.7978	0.9818	0.9656	0.8031	0.9839
DEU-Net [16]	0.8037	0.9821	0.9661	0.8037	0.9812
SA-UNet [19]	0.8573	0.9835	0.9755	0.8153	**0.9905**
IterNet [34]	0.7969	**0.9881**	**0.9760**	0.8072	0.9899
MAU-Net [9]	0.7798	0.9822	0.9620	0.8037	0.9791
SCS-Net [35]	0.8365	0.9839	0.9744	-	0.9867
HT-Net	**0.8477**	0.9834	0.9745	**0.8154**	0.9898

**Table 6:** Comparison experiments based on STARE.

Methods	Se	Sp	Acc	F1	AUC
U-Net [7]	0.8151	0.9887	0.9756	0.8350	0.9908
DUNet [8]	0.8315	0.9858	0.9694	-	0.9905
DEU-Net [16]	0.8204	0.9840	0.9696	0.8247	0.9869
PCANet [33]	0.8098	0.9896	0.9758	0.8371	0.9886
IterNet [34]	0.7715	**0.9919**	0.9782	0.8146	0.9915
MAU-Net [9]	0.7536	0.9808	0.9581	0.7826	0.9721
SCS-Net [35]	0.8207	0.9739	0.9736	-	0.9877
HT-Net	**0.8478**	0.9870	**0.9765**	**0.8459**	**0.9928**

**Table 7:** Cross-training evaluation based on DRIVE and STARE.

Methods	Se	Sp	Acc	F1
STARE (train) -> DRIVE (test)				
TSNet [36]	0.7014	0.9802	0.9444	0.9568
JSPW-Net [37]	**0.7292**	0.9815	0.9494	0.9599
HT-Net	0.7019	**0.9913**	**0.9659**	**0.9825**
DRIVE (train) -> STARE (test)				
TSNet [36]	0.7319	**0.9840**	0.9580	0.9678
JSPW-Net [37]	0.7211	0.9840	0.9569	0.9708
HT-Net	**0.8580**	0.9733	**0.9648**	**0.9840**

## Data Availability

We use three publicly available fundus image datasets to evaluate our proposed segmentation network. They are the DRIVE dataset, CHASE-DB1 dataset, and STARE dataset; the URLs of these datasets are http://www.isi.uu.nl/Research/Databases/DRIVE/ (accessed on 30 May 2022), https://blogs.kingston.ac.uk/retinal/chasedb1/ (accessed on 30 May 2022), and https://cecas.clemson.edu/~ahoover/stare/ (accessed on 1 June 2022), respectively.
